# Large-Scale Evidence for the Effect of the *COLIA1* Sp1 Polymorphism on Osteoporosis Outcomes: The GENOMOS Study

**DOI:** 10.1371/journal.pmed.0030090

**Published:** 2006-02-21

**Authors:** Stuart H Ralston, André G Uitterlinden, Maria Luisa Brandi, Susana Balcells, Bente L Langdahl, Paul Lips, Roman Lorenc, Barbara Obermayer-Pietsch, Serena Scollen, Mariona Bustamante, Lise Bjerre Husted, Alisoun H Carey, Adolfo Diez-Perez, Alison M Dunning, Alberto Falchetti, Elzbieta Karczmarewicz, Marcin Kruk, Johannes P. T. M. van Leeuwen, Joyce B. J. van Meurs, Jon Mangion, Fiona E. A McGuigan, Leonardo Mellibovsky, Francesca del Monte, Huibert A. P Pols, Jonathan Reeve, David M Reid, Wilfried Renner, Fernando Rivadeneira, Natasja M. van Schoor, Rachael E Sherlock, John P. A Ioannidis, for the GENOMOS investigators

**Affiliations:** **1**Rheumatic Diseases Unit, University of Edinburgh, Western General Hospital Edinburgh, Edinburgh, United Kingdom; **2**Department of Medicine and Therapeutics, University of Aberdeen Medical School, Aberdeen, United Kingdom; **3**Department of Internal Medicine, Erasmus MC, Rotterdam, Netherlands; **4**Department of Internal Medicine, University of Florence, Florence, Italy; **5**Department of Genetics, University of Barcelona, Barcelona, Spain; **6**Department of Endocrinology, Aarhus Sygehus, Aarhus, Denmark; **7**Institute for Research in Extramural Medicine, VU University Medical Center, Amsterdam, Netherlands; **8**Department of Biochemistry and Experimental Medicine, The Children's Memorial Health Institute, Warsaw, Poland; **9**Department of Endocrinology and Nuclear Medicine, University of Graz, Graz, Austria; **10**Strangeways Research Laboratory, Cambridge University, Cambridge, United Kingdom; **11**Oxagen Limited, Abingdon, United Kingdom; **12**Hospital del Mar-IMIM, Universitat Autònoma de Barcelona, Barcelona, Spain; **13**Clinical and Molecular Epidemiology Unit, Department of Hygiene and Epidemiology, University of Ioannina School of Medicine and Biomedical Research Institute, Foundation for Research and Technology-Hellas, Ioannina, Greece; Guy's HospitalUnited Kingdom

## Abstract

**Background:**

Osteoporosis and fracture risk are considered to be under genetic control. Extensive work is being performed to identify the exact genetic variants that determine this risk. Previous work has suggested that a G/T polymorphism affecting an Sp1 binding site in the *COLIA1* gene is a genetic marker for low bone mineral density (BMD) and osteoporotic fracture, but there have been no very-large-scale studies of *COLIA1* alleles in relation to these phenotypes.

**Methods and Findings:**

Here we evaluated the role of *COLIA1* Sp1 alleles as a predictor of BMD and fracture in a multicenter study involving 20,786 individuals from several European countries. At the femoral neck, the average (95% confidence interval [CI]) BMD values were 25 mg/cm ^2^ (CI, 16 to 34 mg/cm ^2^) lower in TT homozygotes than the other genotype groups ( *p* < 0.001), and a similar difference was observed at the lumbar spine; 21 mg/cm ^2^ (CI, 1 to 42 mg/cm ^2^), ( *p* = 0.039). These associations were unaltered after adjustment for potential confounding factors. There was no association with fracture overall (odds ratio [OR] = 1.01 [CI, 0.95 to 1.08]) in either unadjusted or adjusted analyses, but there was a non-significant trend for association with vertebral fracture and a nominally significant association with incident vertebral fractures in females (OR = 1.33 [CI, 1.00 to 1.77]) that was independent of BMD, and unaltered in adjusted analyses.

**Conclusions:**

Allowing for the inevitable heterogeneity between participating teams, this study—which to our knowledge is the largest ever performed in the field of osteoporosis genetics for a single gene—demonstrates that the *COLIA1* Sp1 polymorphism is associated with reduced BMD and could predispose to incident vertebral fractures in women, independent of BMD. The associations we observed were modest however, demonstrating the importance of conducting studies that are adequately powered to detect and quantify the effects of common genetic variants on complex diseases.

## Abstract 2

A large collaborative European study finds only weak links between a much studied potential genetic risk factor and bone mineral density or fracture risk.

## Introduction

Osteoporosis is a common disease with a strong genetic component, characterized by reduced bone mineral density (BMD) and an increased risk of fragility fractures. A large number of studies have been conducted on potential associations between candidate gene polymorphisms and various osteoporosis-related phenotypes, such as BMD, quantitative ultrasound properties of bone, and risk of fragility fractures [ 1]. Most of these studies were underpowered to detect the modest effects that are expected for genetic susceptibility to a complex disorder such as osteoporosis [ 2]. Moreover, the results that have arisen from many studies have not been replicated [ 3, 4], or when large-scale evidence has been obtained, results have been observed that were different to those observed in initial studies [ 5]. The *COLIA1* gene, which encodes the alpha I chain of type 1 collagen, is one of the most extensively studied candidate genes for susceptibility to osteoporosis. A single nucleotide polymorphism affecting an Sp1 binding site within a key regulatory region of *COLIA1* [ 6] has previously been reported to be associated with BMD and susceptibility to osteoporotic fracture, particularly vertebral fractures [ 7– 9]. Preclinical studies have indicated that the *COLIA1* Sp1 polymorphism is a functional variant that affects collagen gene regulation and bone quality [ 10, 11]. In keeping with this, the association with fracture in many clinical studies has been found to be independent of BMD [ 7] and of greater magnitude than would be expected from the reported genotype-specific differences in BMD [ 8]. Although the data linking *COLIA1* Sp1 genotype to osteoporosis is ostensibly convincing and has been supported by meta-analyses [ 8, 9, 11], retrospective meta-analysis may yield misleading results due to publication bias or other confounding factors [ 12]. Given the potential importance of the *COLIA1* Sp1 polymorphism as a genetic marker of osteoporosis outcomes on a population level, we set out to generate large-scale evidence for these associations in a collaborative study involving 26,242 individuals from several European centers within the framework of the Genetic Markers for Osteoporosis (GENOMOS) consortium.

## Methods

### Study Cohorts

The GENOMOS project is large-scale study of several candidate gene polymorphisms in relation to osteoporosis-related outcomes in individuals drawn from several European centers [ 5].This report includes seven of the eight cohorts that were included in a meta-analysis of *ESR1* gene polymorphisms [ 5], with additional individuals from Austria (Graz), Netherlands (Amsterdam), and Poland. One of the previously studied cohorts (the Danish Osteoporosis Study) was not included in this analysis because of potential coding errors of some samples. Two of the participating teams (Rotterdam and Barcelona) provided additional fracture data on individuals who had been reported upon previously. For three teams (the European Prospective Osteoporosis Study [EPOS], Aarhus, and Florence), additional fracture and genotype data were provided, and for one team (Aberdeen), the fracture database was updated and validated further compared with the previous GENOMOS analysis on estrogen receptor alpha gene polymorphisms [ 5].

We refer to previous publications on the characteristics of four longitudinal cohorts (the Rotterdam study [ 13], the Aberdeen Prospective Osteoporosis Study (APOSS) [ 14], the Longitudinal Aging Study Amsterdam (LASA) [ 15], and EPOS [ 16]). Three cross-sectional studies of unrelated individuals (Aarhus, Barcelona, and Florence) have previously been described [ 5] as has a cross-sectional family-based study (Familial Osteoporosis Study [FAMOS] run by the company Oxagen) [ 17]. We also included data from two new populations that were not part of our previous meta-analysis. These are the cross-sectional European Polish Osteoporosis Study [EPOLOS] study, which is a population-based study in Poland recruiting men and women of continental European ancestry aged 20–80 y with exclusion criteria of pregnancy, cancer, obesity (weight >100 kg), and fractures occurring during the year prior to enrolment. EPOLOS fracture cases and age- and sex-matched controls were included and analyzed as part of the EPOS cohort. Data were also included from a study coordinated in Graz comprised of individuals from a population-based study of normative ranges for BMD in healthy men and women in Austria aged 21–76 y, as described [ 18]. In addition, the Graz team collected data from a study of elderly nursing homes residents (average age 84 y) in Eastern Austria.

All participating teams contributed information on sex, age, height, weight, *COLIA1* Sp1 genotype, BMD at lumbar spine and femoral neck (in mg/cm ^2^), and (for women) menopausal status and use of hormone replacement therapy. All teams contributed information on fracture although this information had been gathered differently by the participating centers. We originally assembled information as collected by each team and then made efforts to standardize definitions of fracture where possible.

### Prevalent Fractures

In Aarhus, Rotterdam, and Barcelona, fractures that had occurred at, or before the time of enrolment had been validated by radiographic documentation. In Graz, Amsterdam, Barcelona, EPOS, Florence, and Oxagen, fractures had been ascertained on the basis of clinical history or questionnaire. All vertebral fractures had been documented by radiographs with the exception of Aberdeen, where diagnosis was based on questionnaire. In the Amsterdam, Florence, and Oxagen cohorts, fractures occurring at any time of life were counted; for Aberdeen, fractures occurring under age 18 y were excluded; for EPOS and Graz, fractures occurring under age 20 y were excluded; for Barcelona, fractures occurring under age 45 y were excluded; whereas for Aarhus and Rotterdam, only vertebral fractures documented radiographically at the time of enrolment were counted. Fractures of the hands, fingers, toes, feet, face, and skull were excluded in Barcelona. In Graz, fractures of the hands, face, skull, and clavicle were excluded. High-trauma fractures were excluded upfront in Barcelona, EPOS, and Florence as part of the original design of these studies.

### Incident Fractures

For the longitudinal studies, data on incident fractures that had occurred during the period of follow up were available, and only fractures that could be validated by medical records, scrutiny of original radiographs, or radiologist reports were included, except for EPOS where fractures were documented by an interviewer-completed questionnaire. All incident vertebral fractures were validated by radiographs.

### Ethical Considerations

All participating centers have received institutional review board and/or ethics committee approval according to their local regulations, and informed consent has been obtained according to the requirements of each center.

### Genotyping

Genotyping for the *COLIA1* Sp1 G to T polymorphism (dbSNP rs1800012) was performed using various techniques, including Taqman (EPOS, EPOLOS, Graz, and Rotterdam), pyrosequencing (Oxagen), restriction fragment length polymorphism (RFLP) (Amsterdam and Barcelona), and DNA sequencing (Aberdeen). Genotyping for the Florence and Amsterdam cohorts was performed in Rotterdam, and genotyping for the Aarhus cohort was performed in Aberdeen. Standardization between centers was evaluated by blinded genotyping of 50 randomly selected samples by all centers. The results were evaluated by the coordinating center in Rotterdam, and any discrepancies in the reference plate were reported in order to improve calling of genotypes. Genotyping of the reference plate was repeated again, and centers had to switch genotyping techniques if they were still generating more than 5% errors (more than two errors in 50) in the reference plate (one center changed from RFLP to Taqman). After this, full cohort genotypes were generated in all centers. In addition, each center checked their own cohort genotyping afterwards by re-genotyping at least 5% of their samples with random selection, and fewer than 1% discrepancies were observed for each cohort.

### BMD Measurements

BMD was assessed by dual-energy X-ray absorptiometry using Hologic bone densitometers (Hologic, Bedford, Massachusetts, United States) in Aarhus, Amsterdam, Barcelona, Florence, and Graz; Norland XR26 and XR36 densitometers (Norland, Cooper Surgical, Trumbull, Connecticut, United States) in Aberdeen; and a Lunar DPX-L or DPX densitometer (Lunar, GE Medical Systems, Madison, Wisconsin, United States) in Rotterdam and EPOLOS. For the multicenter EPOS and FAMOS studies, a variety of devices were used and cross-calibrated with the European Spine Phantom. Details of these devices and the validation procedure for cross-calibration in the FAMOS study have been described [ 19]. Syntheses of BMD data across studies always include also a study effect that would account both for genuine differences in populations and potential systematic differences between these devices. The results of the meta-analysis for BMD are interpreted with emphasis on the BMD differences between the contrasted genotypes and not on the absolute BMD values.

### Outcomes

The main outcomes assessed were BMD of the lumbar spine and femoral neck; all fractures (as defined by each cohort); and vertebral fractures as documented by clinical or morphometric criteria [ 20]. To take account of differences in methods of diagnosis and definition of fractures across teams, we also performed sensitivity analyses using standardized criteria. These analyses were limited to incident fractures; incident vertebral fractures; and low/no trauma fractures (excluding fractures occurring with high trauma, as assessed by the circumstances in which they had occurred and/or their location). Information on low/no-trauma fractures was available from all teams except Aberdeen and Graz. For all analyses, data in each team were first split according to sex. In all studies, participants were unrelated with the exception of the FAMOS study, of which we used only one randomly selected person per pedigree for our analysis. Sensitivity analysis using all FAMOS participants yielded largely similar results (not shown).

### Analyses

Analysis of variance (ANOVA) considered all possible genotypes and used the study designation as random factor. Adjusted analyses were also performed in which we included age, height, weight, and (for women) hormone replacement therapy and menopausal status as covariates. Estimated marginal means in each genotype group were compared.

We estimated the unadjusted mean BMD and standard deviation in each study for each genotype of interest. We then synthesized BMD differences between genotype contrasts across studies, using fixed and random effects general variance models [ 21]. Between-study heterogeneity was assessed by the chi-square–based *Q* statistic (Cochran's *Q,* considered significant for *p* < 0.10). Random effects models incorporate the between-study heterogeneity and allow for a different effect in each population [ 21]. In the absence of between-study heterogeneity, fixed and random effects are similar. Unless reported otherwise, random effects are presented.

For fractures, we used the odds ratio (OR) as the metric of choice, given the case-control design of some participating teams. First, we investigated per allele (co-dominant) models for all fracture outcomes. Recessive and dominant models were also assessed. In each analysis, ORs were evaluated for between-study heterogeneity using the *Q* statistic (considered significant for *p* < 0.10) and synthesized with fixed (Mantel-Haenszel) and random (DerSimonian and Laird) effects methods [ 21]. Unless stated otherwise, random effects are presented. Adjusted logistic regression analyses were also performed stratified per study and sex (fixed effects) and considering age, height, weight; hormone replacement therapy, and menopausal status for women; and further adjustment for BMD whenever a potential effect was seen, in order to examine whether this was explained by differences in BMD.

Analyses were conduced in SPSS 12.0 (SPSS, Chicago, Illinois, United States), and Meta-Analyst (Joseph Lau, Boston, Massachusetts, United States). *p*-Values are two-tailed. Exact tests for Hardy-Weinberg equilibrium used the GENEPOP program ( http://wbiomed.curtin.edu.au/genepop/) [ 22]. All genotype data per participating center and sex were consistent with Hardy-Weinberg equilibrium.

## Results

### Clinical Data

Data were gathered on 26,242 individuals (18,405 women), and 23,926 participants were analyzed (16,936 women) after selecting only one participant for each FAMOS pedigree ( Table 1). Of the 23,926 participants, data on lumbar spine BMD, femoral neck BMD, all fractures, and vertebral fractures were available on 16,739, 17,133, 23,309, and 18,227 participants, respectively. There were 6,067 individuals with any fracture, 2,088 with vertebral fractures, 2,407 with incident fractures (412 had incident vertebral fractures), and 3,743 with low/no-trauma fractures. Standardized data on Sp1 genotypes were obtained in 20,786 of the 23,926 participants based on sample availability ( Table 1).

**Table 1:**
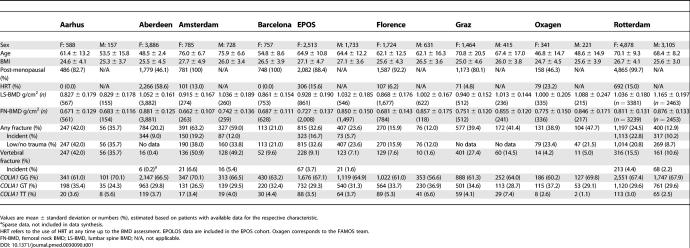
Relevant Clinical Characteristics of the GENOMOS Cohort

### Association between *COLIA1* Alleles and BMD

**Figure 1:**
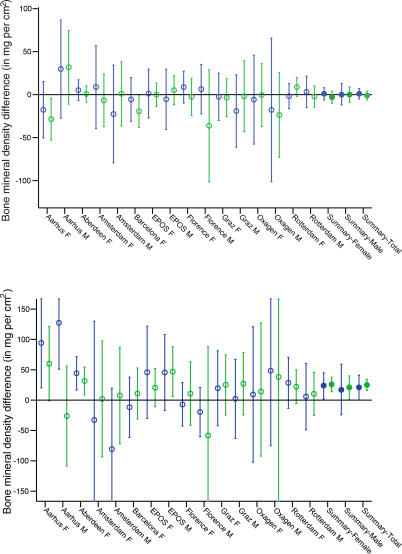
Association between *COLIA1* Alleles and BMD Differences in BMD (in mg/cm ^2^) for the contrasts of GG homozygotes versus GT heterozygotes are shown in the top panel and those for GG and GT combined versus TT homozygotes in the bottom panel. For each study, the point estimates and 95% CIs for the differences in BMD in the lumbar spine (blue) and femoral neck (green) are shown. The figures are purposely drawn putting data on the two skeletal sites side by side in each center for comparison. Summary estimates of the differences and their 95% CIs are given by random effects models for male (M), female (F), and all participants (total). Fixed effects estimates are very similar (not shown). Filled circles represent summary estimates.

The Sp1 polymorphism was associated with BMD both at the lumbar spine ( *p* = 0.039) and femoral neck ( *p* < 0.001) using unadjusted ANOVA ( Table 2). A comparison of marginal means showed that TT homozygotes had significantly lower BMD values than GT heterozygotes at the lumbar spine ( *p* = 0.011) and at the femoral neck ( *p* < 0.001), and lower BMD values than GG homozygotes at the lumbar spine ( *p* = 0.017) and the femoral neck ( *p* < 0.001). There was no difference in BMD values at either site between GG homozygotes and GT heterozygotes ( *p* = 0.80 and *p* = 0.83, respectively). The comparison between GG and GT genotype groups is shown in Figure 1, upper panel. The 95% confidence intervals (CIs) show that it is possible to exclude differences between the groups exceeding 7 mg/cm ^2^ at either skeletal site, which approximates to 0.05 BMD z-score units. There was no significant between-study heterogeneity for this comparison at either skeletal site ( *p* > 0.3 for both). The comparison between TT homozygotes and the other genotype groups is shown in Figure 1, lower panel. Homozygotes for the T allele had significantly lower BMD than the GG and GT genotype groups at the lumbar spine: 21 mg/cm ^2^ (95% CI, 1 to 42); and the femoral neck: 25 mg/cm ^2^ (95% CI, 16 to 34). For the analysis of all study participants, there was some heterogeneity between studies for BMD values at the lumbar spine ( *p* = 0.018), but not at the femoral neck ( *p* = 0.6). Sex-specific analyses showed similar results for females at the lumbar spine: 24 mg/cm ^2^ (95% CI, 3 to 46), *p* = 0.17 for heterogeneity, and the femoral neck: 26 mg/cm ^2^ (95% CI, 14 to 38), *p* = 0.9 for heterogeneity. Results for males were not formally significant for the lumbar spine: 18 mg/cm ^2^ (95% CI, −24 to 59) where significant heterogeneity was observed between studies ( *p* = 0.014); but they were significant at the femoral neck: 21 mg/cm ^2^ (95% CI, 1 to 40) and here, there was no heterogeneity between studies ( *p* = 0.2). Analysis of the data using ANOVA with adjustment for covariates yielded similar results (not shown) as the unadjusted analyses.

**Table 2:**
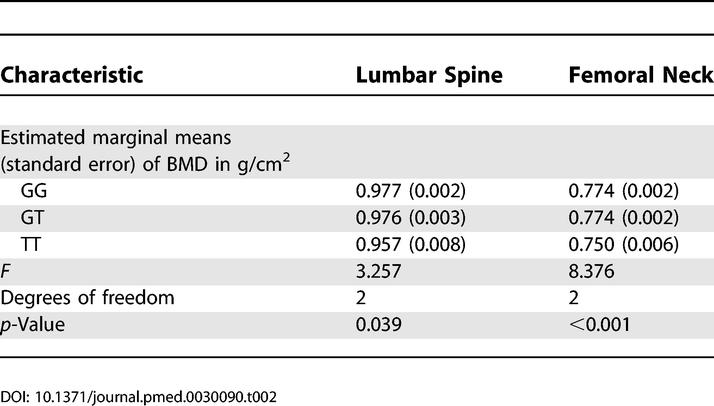
Results of Analysis of Variance for BMD

### Association between *COLIA1* Alleles and Fracture

**Figure 2:**
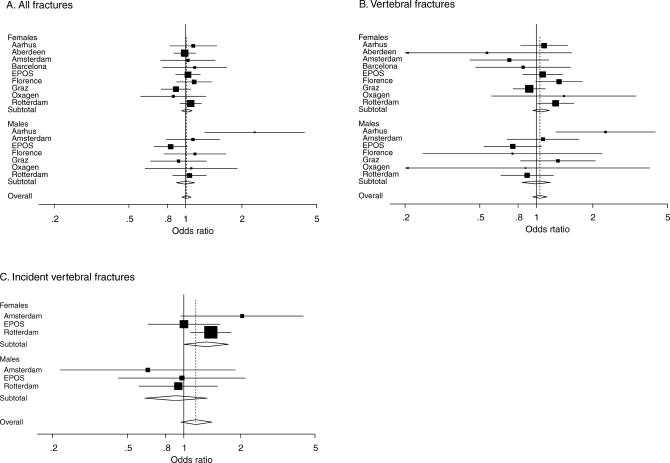
Association between *COLIA1* Alleles and Fractures OR for fractures in co-dominant models (per T allele) for (A) fracture at any site; (B) vertebral fracture; and (C) incident vertebral fracture. Point estimates and 95% CIs are shown for the ORs in each study. Summary estimates of the ORs and their 95% CIs (diamonds) are given by random effects models per gender and for the total database.

There was no significant association between the Sp1 polymorphism and fracture overall, whether the data were analyzed under a co-dominant model ( Figure 2A) or recessive and dominant models of genetic contrasts ( Table 3). The tight 95% CI excluded even modest effects, and results were remarkably similar for both sexes. There was still no evidence of an association when the analysis focused on incident fractures: summary OR per allele 1.02 (CI, 0.94 to 1.12) overall; 1.03, (CI, 0.93 to 1.14) in female participants; 1.02 (CI, 0.84 to 1.23) in male participants; or those in whom high-trauma fractures were excluded: 1.04 (CI, 0.96 to 1.13) overall; 1.06 (CI, 0.97 to 1.15) in female participants; 1.08 (CI, 0.87 to 1.34) in male participants. There was no significant heterogeneity between studies in any analysis with the exception of modest heterogeneity in the subgroup of male particpants in the co-dominant ( *p* = 0.04 for any fracture) and dominant ( *p* = 0.09) models.

**Table 3:**
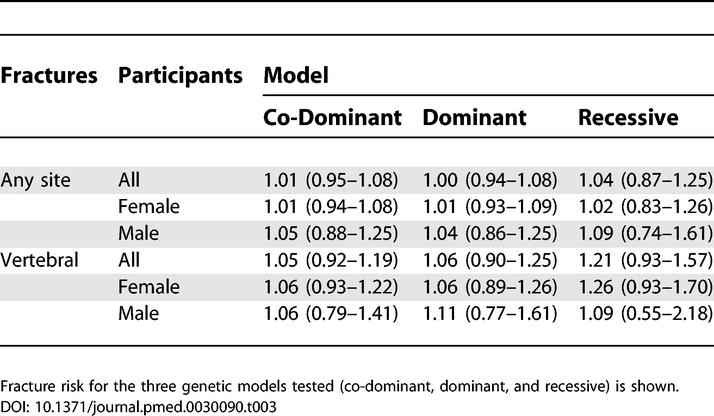
Random Effects ORs (95% CIs) for Fracture Risk

For vertebral fractures, there was no evidence for association in co-dominant ( Figure 2B) or dominant models, whereas there was a non-significant trend for association in the recessive model ( Table 3). There was a borderline significant association for incident vertebral fractures under a co-dominant model ( Figure 2C) particularly in females, where there was a 33% increase in the odds of fracture per allele (OR 1.33 [CI, 1.00 to 1.77], *p* = 0.05). Females with the GT or TT genotype had a 1.40-fold (CI, 1.09 to 1.79) increased risk for vertebral fracture compared with GG homozygotes ( *p* = 0.009, no between-study heterogeneity). The OR was similar for TT homozygotes compared with the other genotype groups, but data were sparse and the result was not significant: OR = 1.33 (CI, 0.69 to 2.58). There was modest heterogeneity in the co-dominant ( *p* = 0.04) and dominant ( *p* = 0.01) models for all vertebral fractures in males, but no heterogeneity in females.

Analyses adjusting for age, height, and weight showed no association between the Sp1 polymorphism and fracture overall (summary OR per allele 1.01 [95% CI, 0.95 to 1.07] *p* = 0.73), and there was no evidence that this differed across different ages ( *p* = 0.22 for interaction between the T allele and age). Adjusting for these factors did not substantially alter the OR estimates for vertebral fractures or incident vertebral fractures (vertebral fractures OR per allele = 1.07 [CI, 0.97 to 1.17], *p* = 0.18; incident vertebral fractures OR per allele = 1.18 [CI, 0.98 to 1.42], *p* = 0.088; and incident vertebral fractures OR for T allele carriers = 1.27 [CI, 1.02 to 1.58], *p* = 0.033). For female participants, the association remained significant after further adjustment for postmenopausal status and hormone replacement therapy use (vertebral fractures OR per allele = 1.10 [CI, 0.98 to 1.23], *p* = 0.11; incident vertebral fractures OR = 1.33 [CI, 1.07 to 1.64], *p* = 0.010; and incident vertebral fractures OR for T alleles carriers = 1.47 [CI, 1.14 to 1.89], *p* = 0.003). There was no significant interaction with age for any of these effects ( *p* > 0.5 for all interaction terms).

Further adjustment for lumbar spine BMD did not considerably alter any of these effects. In both sexes combined, the vertebral fractures OR per allele was 1.08 (CI, 0.96 to 1.22), *p* = 0.19; the incident vertebral fractures OR per allele was 1.24 (CI, 1.00 to 1.53), *p* = 0.048; and the incident vertebral fractures OR for T allele carriers was 1.34 (CI, 1.05 to 1.72), *p* = 0.021.

## Discussion

This multicenter collaborative study of *COLIA1* Sp1 alleles in relation to BMD and fractures is, to our knowledge, the largest ever performed in the field of osteoporosis genetics for a single gene. It indicates that the Sp1 binding site polymorphism of the *COL1A1* gene is associated with BMD at the lumbar spine and femoral neck with a recessive mode of inheritance. The effect size was 21 mg/cm ^2^ at the spine and 25 mg/cm ^2^ at the femoral neck, which corresponds to a difference of about 0.15 BMD z-score units at each site. This differs from previous studies and their meta-analyses where a co-dominant effect of the “T” allele has been observed [ 8, 9]. In this study we found no difference in BMD between GG homozygotes and GT heterozygotes; only for TT homozygotes was the documented association with BMD of a similar magnitude to that reported previously [ 8, 9]. Therefore only a small fraction of the BMD variance would be explained by this Sp1 variant, smaller than previously thought. The association with fracture was also weaker than reported from previous meta-analyses of smaller studies in which dose-response effects were observed with doubling or more of the risk among TT homozygotes [ 8, 9, 11], although the associations in these studies were primarily driven by subjects with vertebral fractures. In this study we found no association with fracture overall or when high-impact traumatic fractures had been excluded. There was a modest association with incident vertebral fractures, particularly in women, which was independent of BMD in adjusted analyses. This effect would translate to an attributable fraction of approximately 10% for incident vertebral fractures in women. Adjustment for multiple comparisons might even invalidate the statistical significance of the modest effect on incident vertebral fractures that we observed. One explanation for the less-impressive genetic effects documented here is that small studies in human genome epidemiology may suffer from selective reporting biases and lack of standardization that can cause inflated effects [ 3, 4, 23, 24]. Even the latest meta-analysis of published information on the *COLIA1* Sp1 which included 26 studies, comprised fewer than 7,000 participants and only 1,326 patients with fractures [ 8]. Importantly, only a small number were TT homozygotes, thereby limiting power to rigorously assess the association between this relatively rare genotype and fracture. Moreover, information could not be standardized across the synthesized published information, which could have led to erroneous results [ 25].

A limitation of our study is that many fractures were ascertained only by clinical history and questionnaires; previous studies in Europe have indicated 11% false-positive and 7% false-negative reporting rates with such ascertainment [ 26]. Another limitation is that different inclusion and exclusion criteria for fracture were used in different cohorts. Neither of these factors is likely to have caused a large bias however, since there is no reason to suppose that individuals with a particular *COLIA1* Sp1 genotype would have been more or less likely to be included according to specific clinical inclusion criteria or because they reported fractures more accurately. Most of the previous studies of fractures in relation to *COLIA1* Sp1 alleles have been in hospital clinic–based populations, focusing on clinical vertebral fracture as the primary outcome. These studies generally did not include data on prior fractures and most had no prospective follow-up. Conversely, in this study we collected all available information on fractures, and we made efforts to analyze fractures with various standardized sensitivity analyses to the greatest extent possible.

A major strength of the GENOMOS project is that it includes over 5-fold more fracture data than all previous reports combined. Other strengths include the fact that the databases have been carefully scrutinized centrally and standardized for definitions, measurements, and genetic contrasts. Moreover, large-scale genotyping was performed for the purposes of this collaborative project. The approach also eliminates the problem of publication bias within the confines of our consortium [ 27]. Nevertheless, our design is not totally immune to biases. Measurement error with non-differential misclassification of genotype or phenotype would tend to diminish the observed ORs. Misclassification in genotyping can seriously affect the results of genetic analysis [ 28], but extensive efforts were made to minimize this in the present study by introduction of strict quality control processes. Although several different densitometers were used in the different cohorts, the same instruments were used in each study center, and all comparisons that focus on genotype differences did so within, rather than between, studies. Misclassifications due to inaccuracies in the clinical history and documentation of fractures are a more serious concern and particularly apply to the data on past fractures. Therefore, the positive association we observed for incident vertebral fractures may reflect to some extent the better accuracy for determining such fractures by standardized radiographic criteria and on a prospective basis.

Previous studies have indicated that the *COLIA1* Sp1 polymorphism is a functional variant that influences DNA protein binding, collagen gene regulation, and bone mineralization [ 10, 11]. The data presented here do not negate these findings, but rather indicate that these effects do not translate into an appreciable clinical association with risk of non-vertebral fractures. Two polymorphisms have been identified in the promoter of *COLIA1* that are in linkage disequilibrium with the Sp1 polymorphisms and are associated with BMD [ 29, 30] and may affect gene transcription [ 31]. It is possible that these and other polymorphisms might interact with the Sp1 variant to influence bone mass and fracture risk in specific populations, but further studies will be needed to clarify this issue. We cannot exclude the possibility that other variants may have larger effects than Sp1.

Our results indicate that large-scale studies are needed to quantify the true effect size of genetic polymorphisms that have been implicated in the pathogenesis of complex common diseases such as osteoporosis. Consortia with standardized large-scale genotyping may place into context the likely effects of multigenetic inheritance.

### Accession Numbers

The SNP database ( http://www.ncbi.nlm.nih.gov/SNP/) single nucleotide polymorphism discussed in this paper is a G/T polymorphism (rs1800012) referenced to the *COLIA1* gene with the GenBank ( http://www.ncbi.nlm.nih.gov/Genbank) accession number J03559.

**Patient Summary**

**Background**

Osteoporosis is a condition characterized by subtle deterioration of bone tissue leading to decreased bone mass and to bone fragility. Doctors often measure a person's bone mineral density to see if he or she has osteoporosis. The major causes of osteoporosis are poor building of bone mass during adolescence and accelerated bone loss after middle age, especially in women (as a result of menopause). Both bone building and bone loss are regulated by genetic and environmental factors. Osteoporosis is thought to be a “complex disease,” with variations in a number of different genes and environmental factors affecting an individual's risk. Researchers have identified a number of genes that are good candidates to play a role in osteoporosis. One such gene is called *COL1A1*. It contains the genetic information to make collagen alpha, a major component of bone and cartilage. Different forms of the *COL1A1* gene have been identified. (Most, if not all human genes, exist in different forms.) One relatively common variant, which might affect bone quality based on experiments in human bone tissue, has been studied intensely in the past. At a particular position in the gene, two versions exist: the more common G version and the T version. Because humans have two copies of the *COL1A1* gene, they can have two G versions, one G and one T version, or two T versions.

**Why Was This Study Done?**

Several previous studies had examined a possible association between the T version of the *COL1A1* gene and low bone mineral density and fractures, and a number of the studies had found such a link. As a consequence, some scientists have suggested that people should get genetic tests for the high-risk variant, and that those who have it should make sure to get enough calcium and do weight-bearing exercises, ideally during the bone-acquisition phase in adolescence. Other scientists have warned that the evidence that links the T version to a higher risk for osteoporosis is not strong enough to support such action. This study was done to find a conclusive answer.

**What Did the Researchers Do and What Did They Find?**

The authors of this study work in universities and hospitals all over Europe. They have joined forces to do what is likely the largest test for a link between a specific genetic variant and osteoporosis. Each group collected data from patients whom they recruited and examined locally, but they all agreed on rigorous common standards upfront and made sure that those were upheld, so that in the end they were able to combine the data from all the patients. They collected data on fractures, measured bone mineral density, and determined whether an individual had the GG, GT, or TT combination of their two *COL1A1* copies for more than 20,000 participants in the study. They found a modest association between the TT version and lower bone mineral density at the femoral neck and the lumbar spine. They did not find an overall link between the TT combination and fractures. However, they did find a weak association between the T version and vertebral fractures in women.

**What Does This Mean?**

Overall, the effects they saw were more moderate than most of the earlier studies. These effects explained only a small part of the differences in bone mineral densities and fractures between the participants. The researchers found no difference in bone mineral density between people who have one copy of the T variant and those who have two G variants (and only approximately 4% of people have two copies of the T variant). Regarding the fracture association, the researchers estimate that having the T variant would explain at most 10% of the risk of vertebral fractures for women. From these results, it seems clear that genetic testing for this particular variant in isolation would be premature. Researchers need to look at other genes (and possibly other variants in the *COL1A1* gene) before they can predict a substantial fraction of an individual's genetic risk for osteoporosis.

**Where Can I Find More Information Online?**

The following Web sites provide information on osteoporosis.

UK National Osteoporosis Society:

http://www.nos.org.uk/public.asp

MedlinePlus pages for osteoporosis:

http://www.nlm.nih.gov/medlineplus/osteoporosis.html

Patient information pages from the Endocrine Society: http://www.hormone.org/learn/osteoporosis.html

## Abbreviations

BMD: bone mineral density
CI: confidence interval
EPOS: the European Prospective Osteoporosis Study
GENOMOS: Genetic Markers for Osteoporosis
OR: odds ratio
