# Correction: Large-Scale Evidence for the Effect of the *COLIA1* Sp1 Polymorphism on Osteoporosis Outcomes: The GENOMOS Study

**DOI:** 10.1371/journal.pmed.0030223

**Published:** 2006-05-30

**Authors:** Stuart H Ralston, André G Uitterlinden, Maria Luisa Brandi, Susana Balcells, Bente L Langdahl, Paul Lips, Roman Lorenc, Barbara Obermayer-Pietsch, Serena Scollen, Mariona Bustamante, Lise Bjerre Husted, Alisoun H Carey, Adolfo Diez-Perez, Alison M Dunning, Alberto Falchetti, Elzbieta Karczmarewicz, Marcin Kruk, Johannes P. T. M van Leeuwen, Joyce B. J van Meurs, Jon Mangion, Fiona E. A McGuigan, Leonardo Mellibovsky, Francesca del Monte, Huibert A. P Pols, Jonathan Reeve, David M Reid, Wilfried Renner, Fernando Rivadeneira, Natasja M van Schoor, Rachael E Sherlock, John P. A Ioannidis

In *PLoS Medicine*, volume 3, issue 4, DOI: 10.1371/journal.pmed.0030090

Among the listed additional authors, J. da Silva, J. Bruges Armas, and A. Lopes Vaz are from Portugal.

